# P-1765. Identification of Total Oxidant Status and Superoxide Dismutase Activity as Predictors of Chagas Heart Disease in South American Immigrants in Spain

**DOI:** 10.1093/ofid/ofaf695.1935

**Published:** 2026-01-11

**Authors:** Maxim Van Herreweghe, Alba Antequera, Julia Pedreira, Carla Morales, Eduard Solé, Subirà Carme, Estefanía Torrecilla, Teresa de Alba, Claudio Parolo, Jose Muñoz, Andrea Angheben, Nina Hermans, Ralph Huits

**Affiliations:** University of Antwerp, Wilrijk, Antwerpen, Belgium; Barcelona Institute for Global Health (ISGlobal), Barcelona, Catalonia, Spain; Barcelona Institute for Global Health (ISGlobal), Barcelona, Catalonia, Spain; Barcelona Institute for Global Health (ISGlobal), Barcelona, Catalonia, Spain; Hospital Clinic de Barcelona, Barcelona, Catalonia, Spain; Barcelona Institute for Global Health (ISGlobal), Barcelona, Catalonia, Spain; Hospital Clinic de Barcelona, Barcelona, Catalonia, Spain; Barcelona Institute for Global Health (ISGlobal), Barcelona, Catalonia, Spain; Universitat Rovira i Virgili, Barcelona, Catalonia, Spain; Hospital Clinic de Barcelona, Barcelona, Catalonia, Spain; IRCCS Sacro Cuore Don Calabria Hospital, Negrar, Verona, Veneto, Italy; University of Antwerp, Wilrijk, Antwerpen, Belgium; IRCCS Ospedale Sacro Cuore Don Calabria, Verona, Veneto, Italy

## Abstract

**Background:**

Chagas disease (caused by *Trypanosoma cruzi*) is endemic in most Latin American countries and a growing public health concern among immigrants in European countries. The role of oxidative stress (OS; an imbalance between damaging oxidants [ROS] and antioxidant defenses) in acute Chagas disease is well-established. OS is also involved in the pathophysiology of chronic complications, particularly Chagas cardiomyopathy. We compared the association of markers of OS in plasma, serum, urine, erythrocytes and peripheral blood mononuclear cells (PBMCs) with measures of disease severity in South American immigrants and selected controls.

**Figure:**
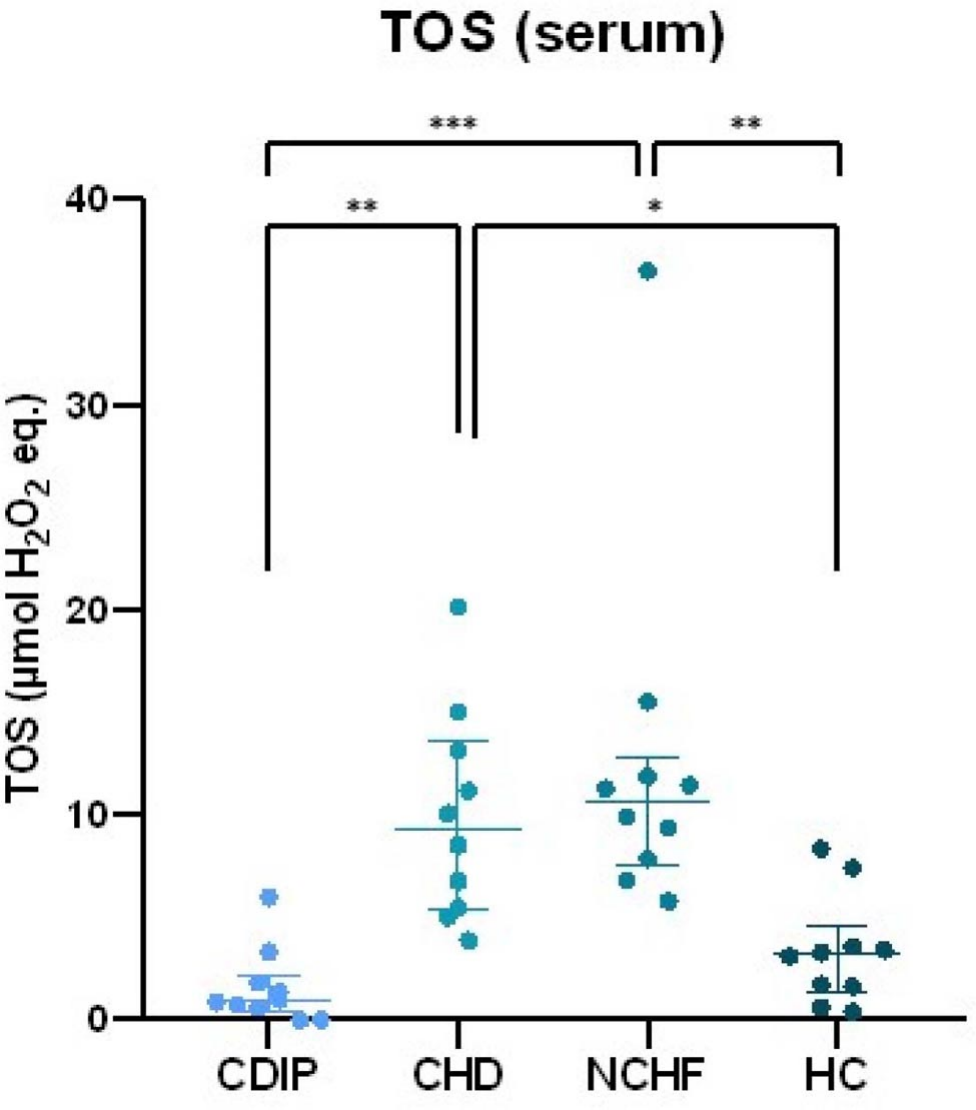
Total Oxidant Status (TOS) levels in serum of the different subject groups. Group comparisons were done using ANOVA testing followed by a Tukey post-hoc test. CDIP = Chagas disease - intermediate phase CHD = Chagas heart disease NCHF = non-Chagas heart failure HC = healthy control. * p < 0.05 ** p < 0.01 *** p < 0.001

**Figure:**
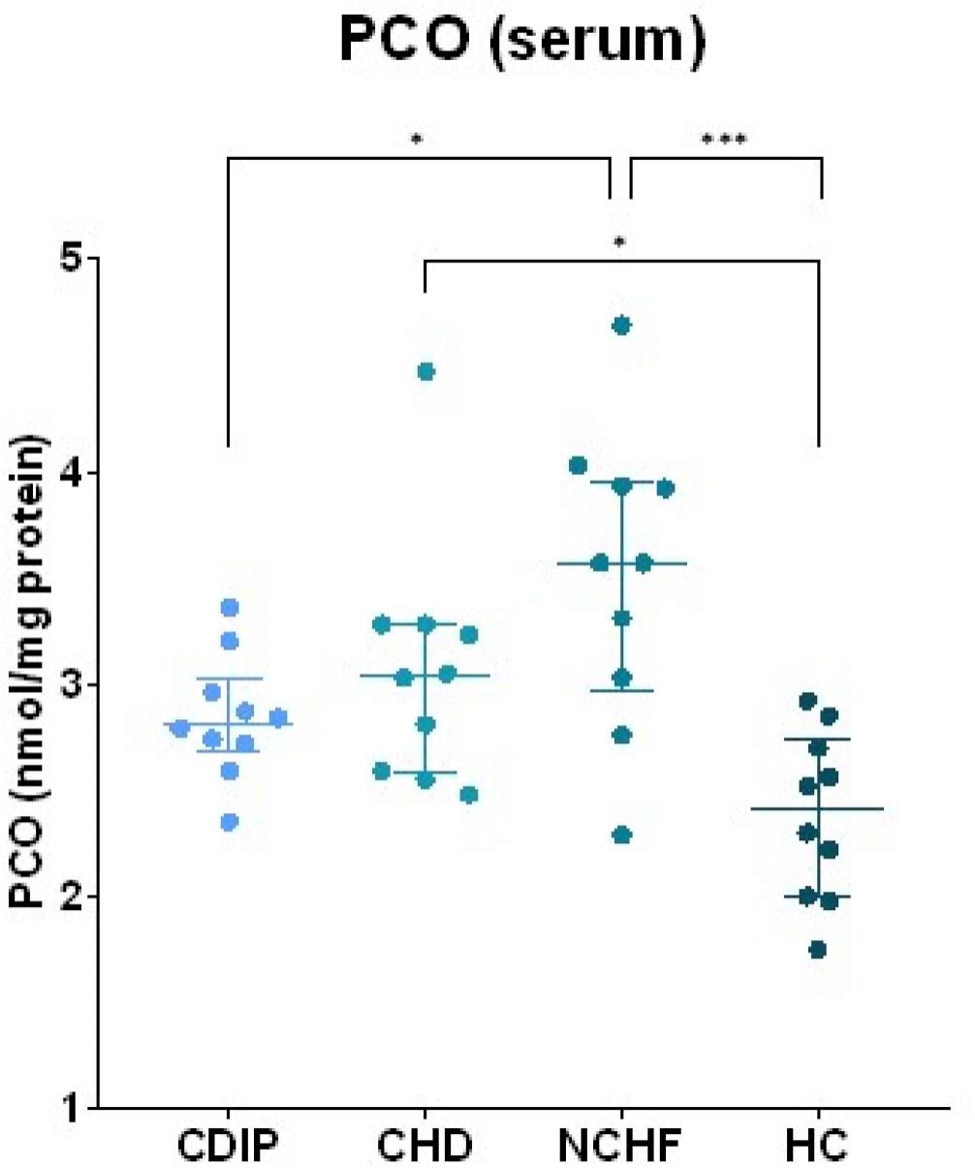
Protein carbonyl (PCO) levels in serum of the different subject groups. Group comparisons were done using ANOVA testing followed by a Tukey post-hoc test. CDIP = Chagas disease - intermediate phase CHD = Chagas heart disease NCHF = non-Chagas heart failure HC = healthy control. * p < 0.05 *** p < 0.001

**Methods:**

We enrolled 4 groups of 10 subjects at the Hospital Clínic de Barcelona (Spain): Bolivian or Ecuadorean immigrants with chronic *T. cruzi* infection in the indeterminate phase (CDIP) or with cardiac damage (CHD), *T. cruzi* seronegative subjects from endemic countries (HC) and seronegative patients with heart failure (NCHF). We measured 10 biomarkers of OS in plasma (CAT_act_, GPx_act_, MDA, SOD_act_), serum (PCO, TAS, TOS), urine (8-OHdG), erythrocytes (GSH) or PBMCs (Nrf2) in duplo. Chronic cardiac damage was classified into stages according to international recommendations adapted to Chagas disease by the American Heart Association. Groups were compared using Kruskal-Wallis or ANOVA followed by post-hoc testing.

**Figure:**
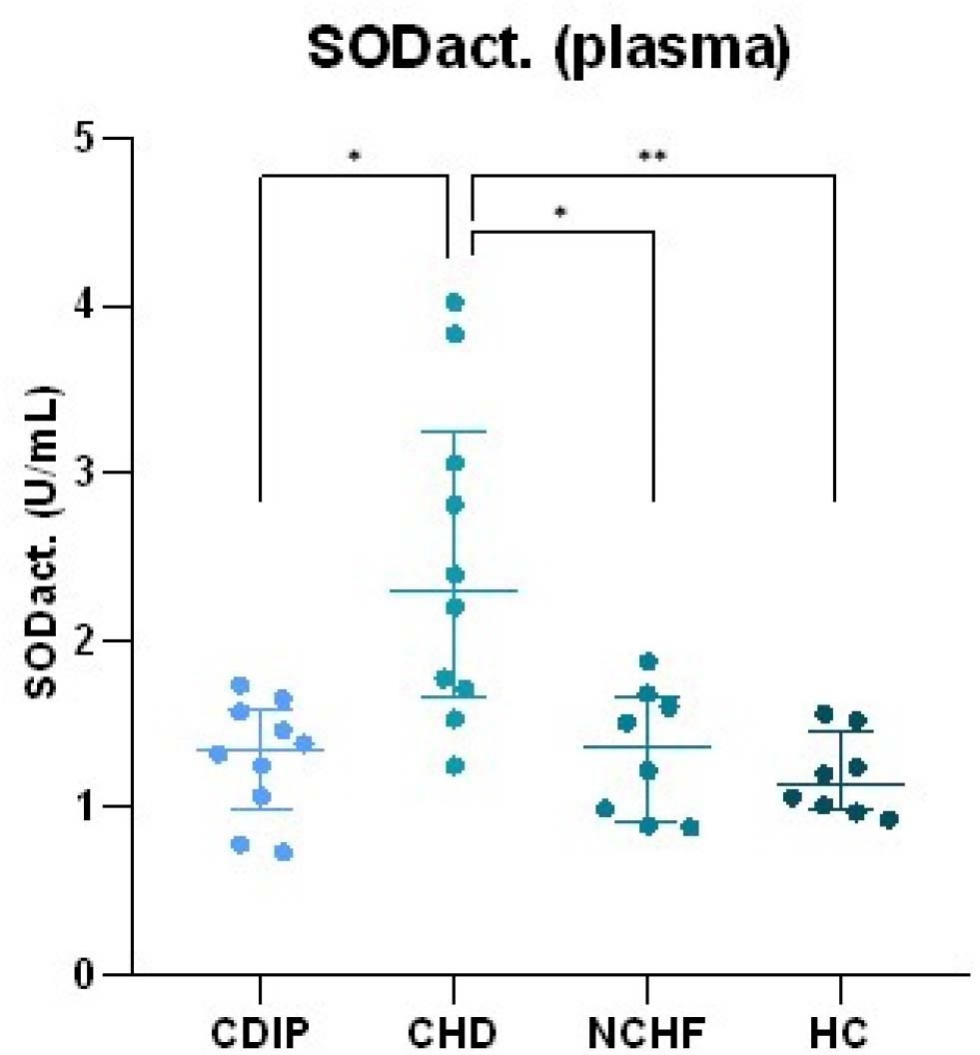
Superoxide dismutase (SOD) activity in plasma of the different subject groups. Group comparisons were done using Kruskal Wallis testing followed by a Dunn's post-hoc test. CDIP = Chagas disease - intermediate phase CHD = Chagas heart disease NCHF = non-Chagas heart failure HC = healthy control. * p < 0.05 ** p < 0.01

**Figure:**
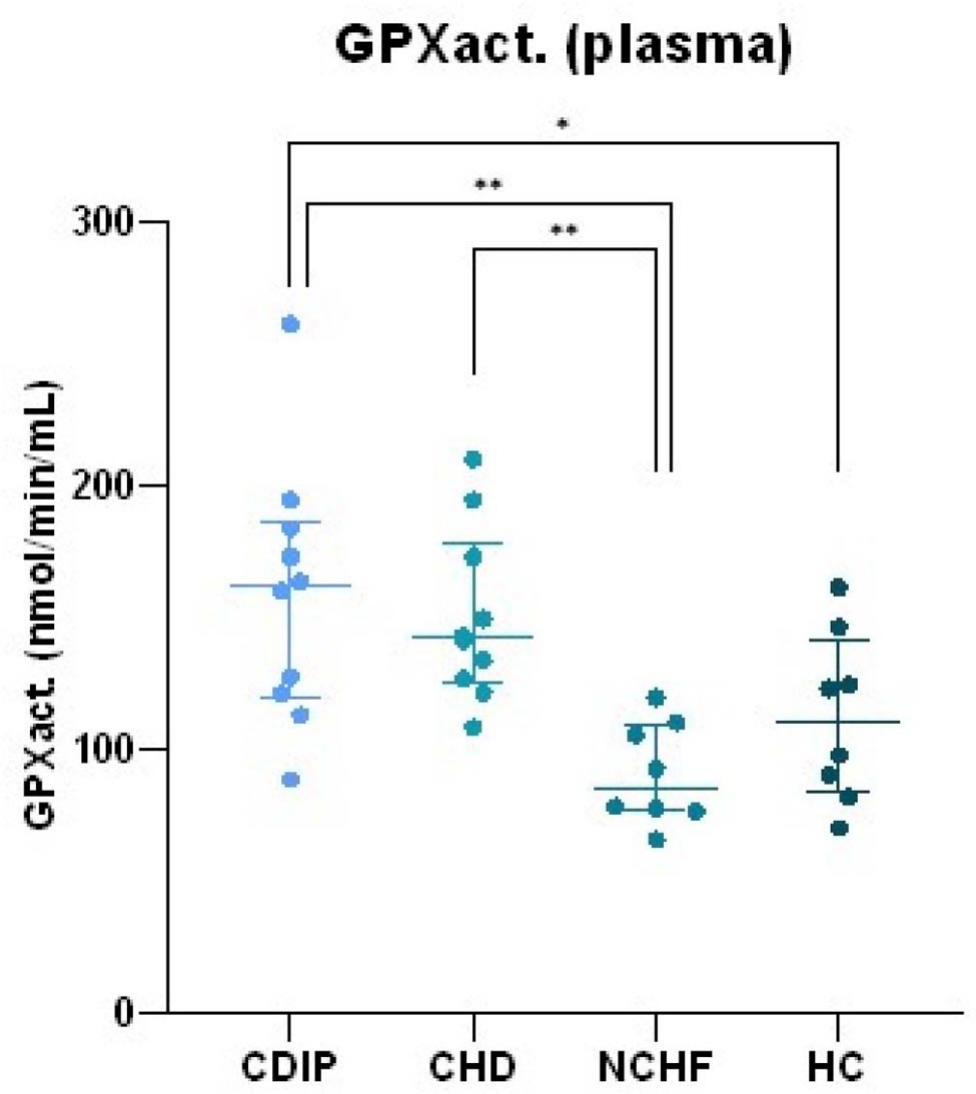
Glutathione peroxidase (GPx) activity in plasma of the different subject groups. Group comparisons were done using ANOVA testing followed by a Tukey post-hoc test. CDIP = Chagas disease - intermediate phase CHD = Chagas heart disease NCHF = non-Chagas heart failure HC = healthy control. * p < 0.05 ** p < 0.01

**Results:**

Mean TOS levels were higher in CHD and NCHF groups compared to HC and CDIP (9.9 and 12.6 vs. 3.4 and 1.6 µmol H_2_O_2_ eq., respectively, shown in image 1). Mean PCO levels were higher in CHD and NCHF compared to HC (3.1 and 3.5 vs. 2.4 nmol/mg protein, image 2). SOD_act_ was significantly increased in CHD compared to all other groups (image 3). Other antioxidant enzymes did not follow this trend, although CDIP had higher mean GPx_act_ than HC and NCHF (159 vs. 112 and 91 nmol/min/mL, image 4). Additionally, extreme values of SOD_act_, TOS, PCO and/or GPx_act_ were associated with sinusal disfunction, atrial arrhythmia, valvulopathy and pacemaker rhythm. These associations differed between CHD and NCHF.

**Conclusion:**

These results suggest that not all biomarkers of OS are altered uniformly, but TOS and SOD_act_ could be useful to differentiate between CDIP and CHD. Selectively upregulated SOD_act_ can be explored as a potential target for treatment options in CHD.

**Disclosures:**

Eduard Solé, MD, Astrazeneca: Advisor/Consultant|Janssen Pharmaceutica: Advisor/Consultant|Novartis: Advisor/Consultant Nina Hermans, PhD, Professor, Tilman: Grant/Research Support

